# Functional Morphology of the Mouthparts of the Adult Mediterranean Fruit Fly, *Ceratitis capitata*

**DOI:** 10.1673/031.008.7301

**Published:** 2008-11-17

**Authors:** Pablo A. Coronado-Gonzalez, S. Vijaysegaran, Alan S. Robinson

**Affiliations:** ^1^ Entomology Unit, FAO/IAEA Agriculture & Biotechnology Laboratory, IAEA aboratories, Seibersdorf, Austria; ^2^ International Centre for the Management of Pest Fruit Flies, Griffith School of Environment, Griffith University, Nathan 4111, Australia

**Keywords:** Tephritidae, proboscis, anatomy, labellar filtering, feeding mechanism

## Abstract

Food-based attractants incorporating an insecticide are an important component of area-wide control programmes for the Mediterranean fruit fly, *Ceratitis capitata* (Wiedemann) (Diptera: Tephritidae). This study was carried out to understand the feeding mechanism of adults of this species. Mouthparts of *C*. *capitata* are similar in general structure to those of another Tephritid genus, *Bactrocera*, and have specific structural modifications that determine what adult flies can ingest. The labellum has a series of fine tube-like structures, called pseudotracheae, on its inner surface. Each pseudotrachea leads from the outer margin of the labellum and ends at the prestomum to the oral opening. The pseudotracheae contain fine micropores about 0.5µm in size. During feeding, the oral opening is never exposed to the feeding substrate but the portions of the opposing labellar lobes proximal to the oral opening are flexed against each other and distal portions of the opposing labellar lobes are opened and pressed flat against the feeding substrate or surface. The prestomal spines at the base of each pseudotrachea interlock to form a barrier across the oral opening. Thus entry of large particles directly into the crop and gut through the oral opening is prevented by flexure of the opposing labellar lobes against each other and the interlocking prestomal spines across the oral opening. Only liquids and suspended particles less than 0.5µm in size are sucked through the micropores into the lumen of the pseudotracheae and then pass into the food canal and into the crop and gut. The pseudotracheae of adult *C*. *capitata*, particularly along the middle portion of the labellum, have prominent blade-like projections that *Bactrocera* do not have. These projections are probably an ancestral condition as they were not observed to use them to abrade the plant or feeding surface as has been reported for species in the Tephritid genus, *Blepharoneura*.

## Introduction

The Mediterranean fruit fly, *Ceratitis capitata* (Wiedemann) is a major pest of fruits and fruiting vegetables, and has been studied extensively because of its economic importance to horticultural production and trade in many parts of the world (Christenson and Foote 1960; White and Elson-Harris 1992). Consequently, a very large volume of information has been amassed on the biology, ecology, behaviour and control of *C*. *capitata* (Robinson and Hooper 1989), but apart from studies by Hanna (1947) and Elzinga and Broce (1986), surprisingly little is known about mouthpart structure and feeding mechanisms in the adult. The structure of the mouthparts has been shown to affect the mode of feeding and the types of food that can be ingested. For example, modifications of the pseudotracheae and prestomal teeth on the labellum enable adult blowflies and *Musca* species to rasp or pierce animal tissue (Graham-Smith 1930), hover flies to ingest nectar and pollen (Gilbert 1981; Schuhmacher and Hoffmann 1982), and lauxaniid flies to feed on fungi (Broadhead 1984). One species of tephritid in the genus *Blepharoneura* uses blade-like modifications of its pseudotracheal ring tips to abrade and feed on plant tissue (Driscoll and Condon 1994). Vijaysegaran et al. (1997), in a comprehensive study on mouthpart structure, feeding mechanisms and natural food sources of adult dacine tephritids, showed that adult *Bactrocera* possess an elaborate labellar filtering mechanism comprising specific structural modifications on the labellum. Combined with a fluid-centred mode of feeding, adult *Bactrocera* flies utilize the labellar filtering mechanism to filter out coarse particles and selectively ingest only fluids, bacteria and particles less than 0.5 µm in size.

Food-based attractants, particularly poisoned protein bait sprays, are an important component in area-wide integrated pest management programmes for *C*. *capitata* (Peck and McQuate 2000) and can even be very effective when incorporated into eradication programmes for *Bactrocera* species (Hancock et al. 2000). Protein baits and other food-based lures have to compete with natural sources of food found in the wild, the identity of which is uncertain that has led to several theories as to what adult fruit flies feed on in nature. The types of foods that *C*. *capitata* can ingest have never been analyzed with regard to mouthpart structure. These investigations were done to examine the structural morphology of the mouthparts of adult *C*. *capitata* with a view towards providing a proper morphological basis for understanding their natural food sources.

## Materials and Methods

### General anatomy of proboscis

The research was carried out using adult flies from the VIENNA 7–98 *C*. *capitata* genetic sexing strain that was mass reared at the FAO/IAEA Agriculture and Biotechnology Laboratory, Seibersdorf, Austria. Improved versions of this strain are now being used in many operational SIT programmes in order to produce males for radiation and release (Robinson et al. 1999).

To clear and expand the labella, 20 newly emerged adult flies of both sexes were anaesthetised with nitrogen in a plastic cage (11×11.5×16.5 cm) for two minutes. They were then immersed in a 10% KOH solution for 24 h and kept at 40°C. Cleared specimens were dried by immersion in ethanol series from 10% to absolute ethanol. The specimens were kept for 15 minutes at each concentration to reach the critical dry point and to prevent the collapse of the labellum. Specimens were then washed and cleared with glacial acetic acid for 15 minutes and immersed in glycerol. They were observed under a Nikon (www.nikon.com) Dissecting Microscope and photographed with a Pentax (www.pentex.com) Camera using Kodak (www.kodak.com) Elite Chrome 200 ASA film.

**Figure 1.**
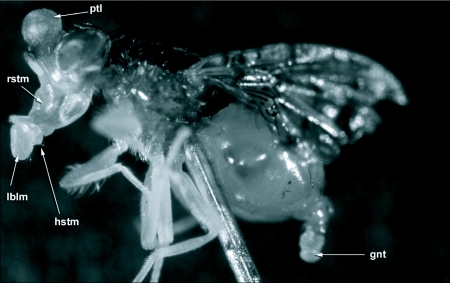
An adult male *Ceratitis* *capitata* injected through the ventral side of the thorax with diethyl ether to cause full extension of the mouthparts. rstm, rostrum; hstm, haustellum; lblm, labellum; ptl, ptilinium and the gnt, genitalia.

A second set of flies was prepared for scanning electron microscopy. Twenty newly-emerged flies were anesthetised with nitrogen and diethyl ether (99.5%) was injected into the haemocoele using a 1ml syringe inserted through the ventral side of the thorax. As the diethyl ether was rapidly evaporating, the negative vapour pressure resulted in extension of the proboscis and full distension of the labellum as well as the ptilinum and genitalia (Figure 1). With the labellum fully extended, the thorax was immediately ligated with a fine nylon thread and specimens were immersed in an Eppendorf vial containing diethyl ether for further drying for 24h. Specimens were then transferred to a 500 ml hermetically sealed glass bottle and a 10 cc syringe was used to extract the air, the purpose of which was to maintain a vacuum to keep the labellum fully extended until the diethyl ether evaporated and specimens were completely dry. Specimens were kept in this manner for 16 h in the bottle at room temperature, after which air was gradually introduced into the bottle to return it to atmospheric pressure. After drying in this manner, the head was removed, cut in different sections and prepared for electron microscopy. Entire or sectioned heads were mounted on copper stubs covered by a layer of liquid silver and specimens were then coated with gold. They were examined with a JEOL- JSM 5200 Scanning Electron Microscope (www.jeol.com) and photographed with a Konica FI-1 Camera (www.konicaminolta.com) using Elite Chrome Kodak100 and Fuji Sensia II film (www.fujifilm.com).

### Adult feeding experiments

To determine the maximum size of solid particles that adult flies can ingest, feeding tests were conducted in which flies were fed particles of various sizes in a suspension of 1% (wt:vol) sucrose or enzymatic yeast hydrolysate. Particles used and their sizes were as follows: (1) pollen grains of *Bellis perennis* (33–38 µm diameter), *Doronicum columnae* (25–34 µm diameter), *Cornus sanguinea* (27–37µm diameter) and *Cardaria draba* (20–27µm diameter); (2) conidiospores of the fungus *Fumagina* sp. (3–5 µm diameter); and (3) non-toxic water colours with particles that ranged in size from <0.5 to 10 µm.

One-week-old *C*. *capitata* fed only sucrose and water from eclosion, were used for each of the three particle sizes tested. They were deprived of food and water for 12 hours prior to feeding them the test solutions. Flies were held singly in glass vials and provided with an excess (one drop) of the test suspension on a glass microscope slide. Flies were allowed to feed until their crops were fully distended and they ceased feeding. They were then anesthesized, the crop was dissected, and its contents were pipetted into a haemocytometer and observed under a microscope (×400). Twenty flies were fed and dissected for each particle size tested.

## Results

### General anatomy of proboscis

**Figure 2.**
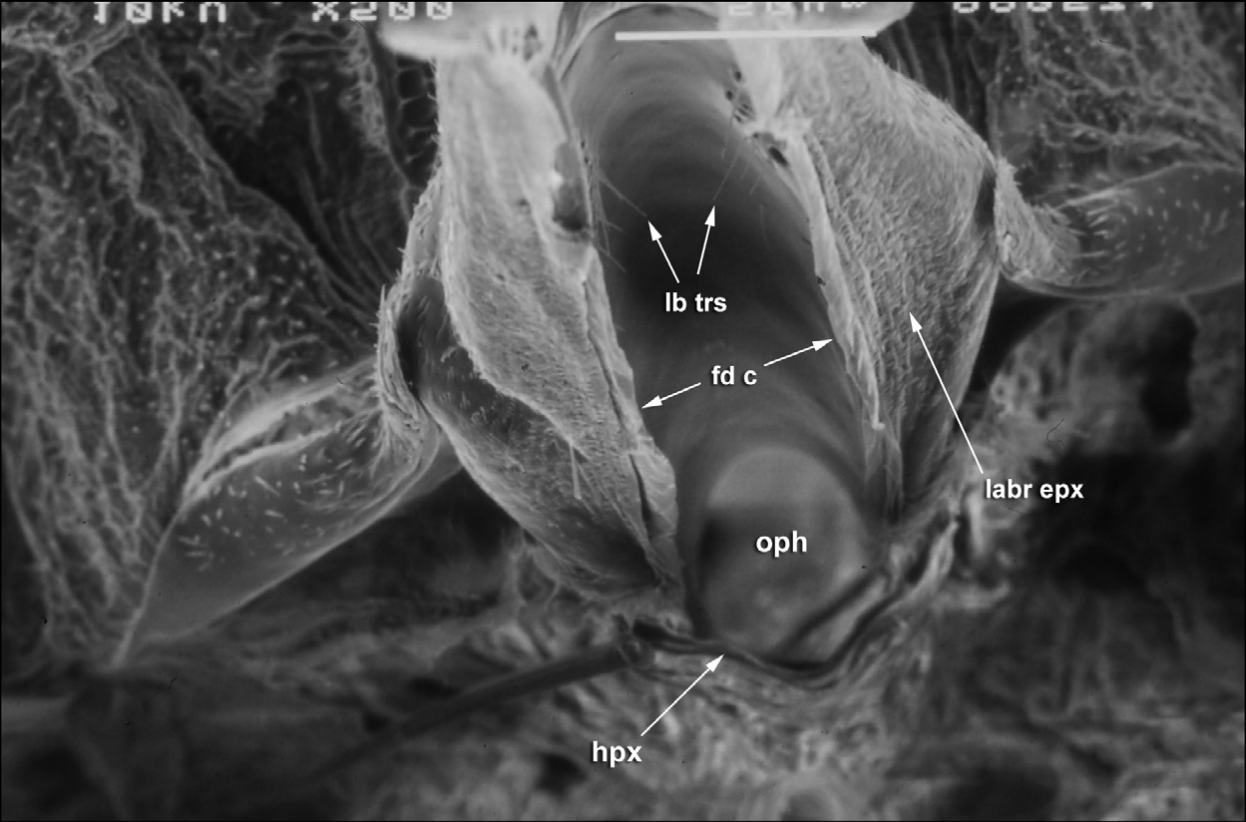
The hypopharynx (hpx) and the labrum epipharynx (labr epx) of *Cerotitis* *capitata*. The diameter of the food canal (fd c) and the opening into the pharynx (oph) is about 35µm and no food particles larger than this can be ingested into the pharynx, hpx, hypopharynx; labr epx, labrum epipharynx; lb trs, labral trichoid sensilla; oph, opening into the pharynx.

**Figure 3.**
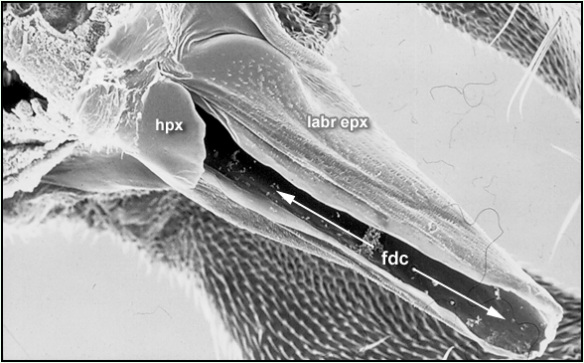
The labrum epipharynx (labr epx) of *Ceratitis* *capitata* to show the highly reduced spade-like hypopharynx (hpx). fdc, food canal; hpx, hypopharynx; labr epx, labrum epipharynx.

The proboscis of *C*. *capitata*, as in other cyclorraphous Diptera, consists of three main parts, a basal rostrum, a median haustellum, and a terminal pair of fleshy oral or labellar lobes (labellum) (Figure 1). The detailed structure of both the rostrum and haustellum in *C*. *capitata* is similar to that of *Bactrocera* and the reader is referred to Figures 1, 2, 3, and 4 in Vijaysegaran et al. (1997) for a clearer understanding of *C*. *capitata* rostrum and haustellum structure. The labellum of *C*. *capitata*, however, has some modifications that are different from *Bactrocera* species and these differences are described in detail in this paper. The terminology of Graham-Smith (1930), McAlpine (1981) and Vijaysegaran et al. (1997) has been adopted to describe *C*. *capitata* mouthpart structure.

The basal rostrum is cone-shaped and it consists of the fulcrum, which is an oval-shaped sclerite with a membranous roof.Its two lateral sides project up and backwards where they join to form the clypeus. It has no distinguishing features and is in general similar in overall structure to that found in other tephritids and muscids. The median haustellum is formed dorsally by the labrum-epipharynx and ventrally by the labium. The tunnel-like space between the labrum-epipharynx and the labium is the food canal. As is typical of Diptera (McAlpine 1981), the labrum and the epipharynx are fused into a single structure called the labrum-epipharynx (Figure 2). The labium-epipharynx is visible as a pointed beak-like structure sitting within the upper folds of the haustellar membrane. The epipharynx is semicircular in cross section and forms the roof of the food canal. The lower portion of the food canal is formed by the haustellar membrane, which is really the labium (see Figure 4 in Vijaysegaran et al. 1997). The structure of the lower portion of the food canal in *C*. *capitata* is different from other Diptera with muscoid type mouthparts, and is similar to that first reported for *Bactrocera* flies by Vijaysegaran et al. (1997). In the blow fly (Graham-Smith 1930) and the house fly (Hewitt 1914), a long blade-like hypopharynx forms the base of the food canal and also houses the salivary canal. Saliva is delivered to the oral opening through a specialized salivary canal. In contrast, the hypopharynx in the *C*. *capitata* (and in *Bactrocera* spp.) is greatly reduced and appears as a short spade-like structure that opens into the upper end of the food canal (Figure 3) (see Figure 3 in Vijaysegaran et al. 1997). Saliva is thus not delivered to the oral opening through a specialized salivary canal as in the blowfly and the housefly. The food canal in *C*. *capitata* ends anteriorly in a vesicle, the prestomum.

**Figure 4.**
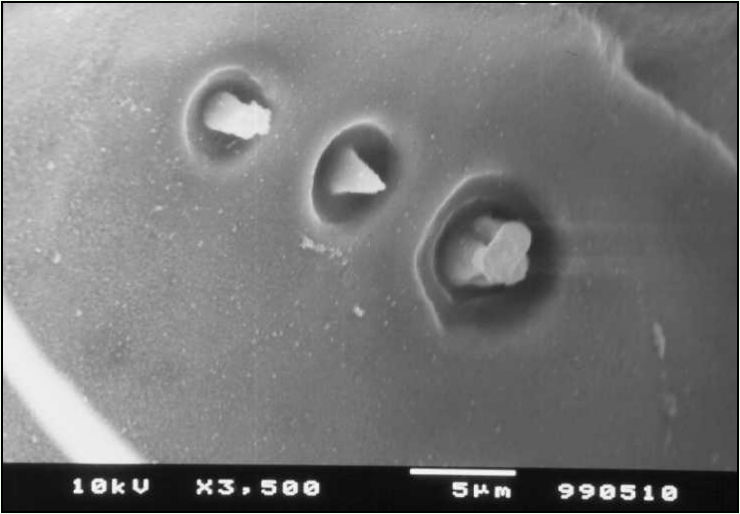
Labral basiconic sensilla of *Ceratitis capitata*.

**Figure 5.**
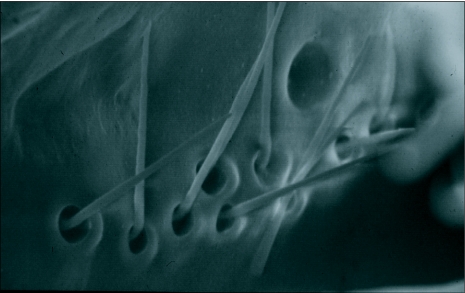
Labral trichoid sensilla of *Ceratitis* *capitata*.

On the inner surface of the labrum-epipharynx are two types of sensilla which occur in paired sensory patches. The portion of the epipharynx proximal to the oral opening, i.e. closer to labellum, has three different labral basiconic sensilla on each sensory patch (Figure 4). Such sensilla are gustatory in function (Rice 1989). About midway along the epiparynx, is a set of 10–12 labral trichoid sensilla on each sensory patch (Figure 5). Such sensilla are tactile fluid-flow in function (Rice 1989). Female *C*. *capitata* have three different labral basiconic sensilla on each sensory patch whereas male Mediterranean fruit flies have only two different labral basiconic sensilla on each sensory patch. The reason for the difference between the number of labral trichoid sensilla on male and female *C*. *capitata* is unknown.

The hypopharynx is hinged to the ventral edge of the hyoid sclerite, which enables articulation for the haustellum to be folded into the rostrum. The opening into the pharynx from the food canal is thus formed by the clypeus dorsally and the hyoid sclerite ventrally (Figure 2) (also see Figure 4A in Vijaysegaran et al. 1997). The opening into the pharynx has a fixed diameter of about 35–40 µm, which critically limits the size of food particles that can be ingested by *C*. *capitata* to particles <40 µm in diameter (Figure 2).

**Figure 6.**
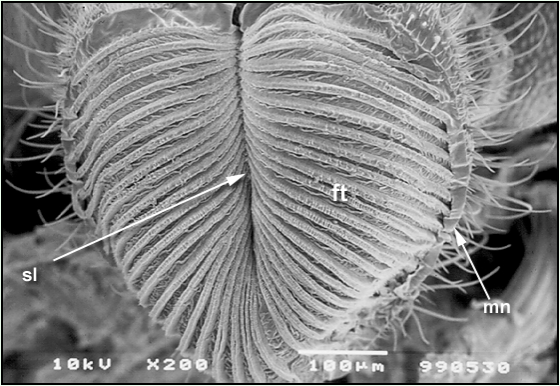
The everted labellum of *Ceratitis* *capitata* showing the labellar pseudotrachea leading from the oral margin (mn) to the oral opening, pt, pseudotracheae, mn, margin of labellar lobe; sl, prestomal sulcus

**Figure 7.**
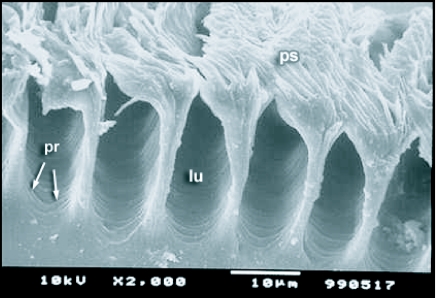
The portion of the labellum of *Ceratitis* *capitata* at the oral opening showing the tube-like structure and the lumen (lu) of the pseudotracheae. Liquids carrying fine particles < 0.5µm flow through the lumen of the pseudotracheae and into the food canal. lu, lumen of the pseudotracheae; ps, prestomal spines; pr, pseudotracheal ring

**Figure 8.**
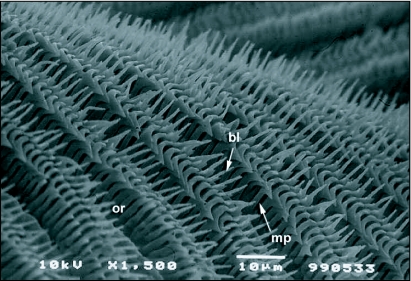
Middle portion of the labellum of *Ceratitis* *capitata* showing rows of pseudotracheae with blade-like modifications of the pseudotracheal rings, bl, blade-like projection of the pseudotracheal ring; mp, micropore; or, opposing pseudotracheal rings.

**Figure 9.**
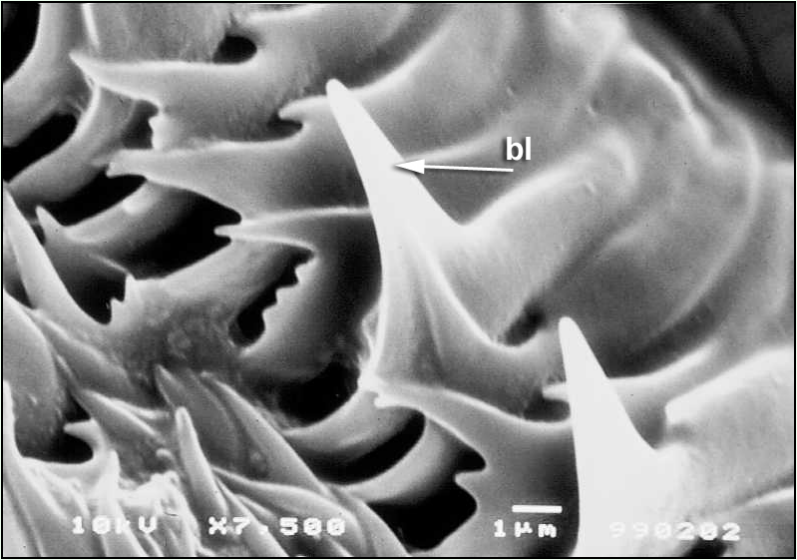
Fine structure of an opposing pseudotracheal ring of *Ceratitis* *capitata* to show the prominent blade-like projections, bl, blade-like projection.

**Figure 10.**
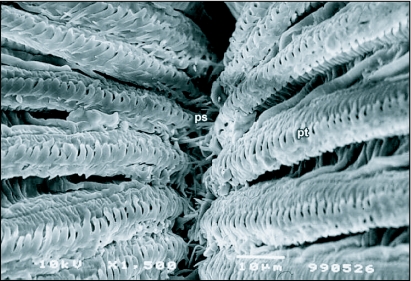
Interlocking prestomal spines (ps) of *Ceratitis* *capitata* that prevent the entry of large food particles into the oral opening, ps, prestomal spines; pt pseudotracheae.

The haustellum gives rise to the oral lobes, which make up the labellum (Figure 6). The terminal labellum consists of two membranous lobes that, when distended, are more or less oval in shape. Each lobe is separated from the other by a vertical space when the proboscis is in a resting position. The inner surface of each labellar lobe has about 20–22 fine tubes, the pseudotracheae, which occasionally merge but most commonly empty into the prestomum. All of the pseudotracheae communicate to the prestomum that is situated between the upper surfaces of the two lobes near the oral opening. Each pseudotrachea is a fine tube-like structure formed by a series of opposing rings. The rings are spaced about 0.5 µm apart and the spaces open into the pseudotracheal lumen (Figure 7). The pseudotracheal rings, particularly those in the middle of the labellum, also bear one or more prominent blade-like projections (Figures 8 and 9). The pseudotracheal rings nearest the oral opening are modified into spines. These spines interlock when the opposing labellar lobes are brought together during feeding and act as a filter that prevent large solid particles from directly entering the pharynx (Figure 10).

### Feeding mechanisms

When exposed to dry or semi solid food, adult flies always regurgitated a copious amount of crop fluids that liquefied and dissolved the food substrate. Food particles and liquids were ingested through the spaces between the pseudotracheal rings and apparently never directly through the oral opening, which has a diameter of about 35–40 µm. Examination of the crop contents of flies fed with particles of different sizes (ranging from <1 µm to >60µm) revealed only a large amount of liquid and particles smaller than 0.5 µm.

The prominent blade-like projections found on the pseudotracheae, particularly in the middle of the labellum (Figures 8 and 9), are presumably used during feeding to scrape the plant surface allowing them to feed on the exuded sap. In feeding experiments, however, scarification of the feeding surface by *C*. *capitata* was not observed. Thus *C*. *capitata*, like *Bactrocera* species, exhibits labellar filtering and ingests only fluids and particles less than 0.5 µm in size (Vijaysegaran et al 1997).

## Discussion

As in the genus *Bactrocera* (Vijaysegaran et al. 1997), the mouthparts of the *C*. *capitata* have specific structural modifications that determine what adult flies can ingest and thus the type of food they feed on in nature. First, like *Bactrocera* species, adult *C*. *capitata* also have a fluid-centered mode of feeding i.e. they feed on dry and semi solid substrate by regurgitating their crop contents to liquefy the substrate and then ingesting the dissolved portion. Regurgitation of crops contents, rather than just salivary secretion, is the manner in which adult tephritids seem to feed. Vijaysegaran et al. (1997) demonstrated that dehydrated adult *Bactrocera*, i.e. without liquid in their crops, were unable to ingest dry and semi-solid substrate. However, when adult flies were rehdyrated, they were immediately able to feed on such substrates. Regurgitation of crop contents did not occur when flies fed on dilute solutions of food where no liquefaction of the substrate was required. In Diptera where saliva is delivered to the oral opening, the hypopharynx is well developed and houses the salivary canal (Hewitt 1914; Graham-Smith 1930). *C*. *capitata* and *Bactrocera* species, however, have a highly reduced hypopharynx, suggesting that the mouthparts are not designed for saliva to be delivered directly to the feeding surface. The reduced hypopharynx has also been reported in other tephritids like *Euaresta* species (Peterson 1916), suggesting that this may be a common condition in the family Tephritidae as a whole.

**Figure 11.**
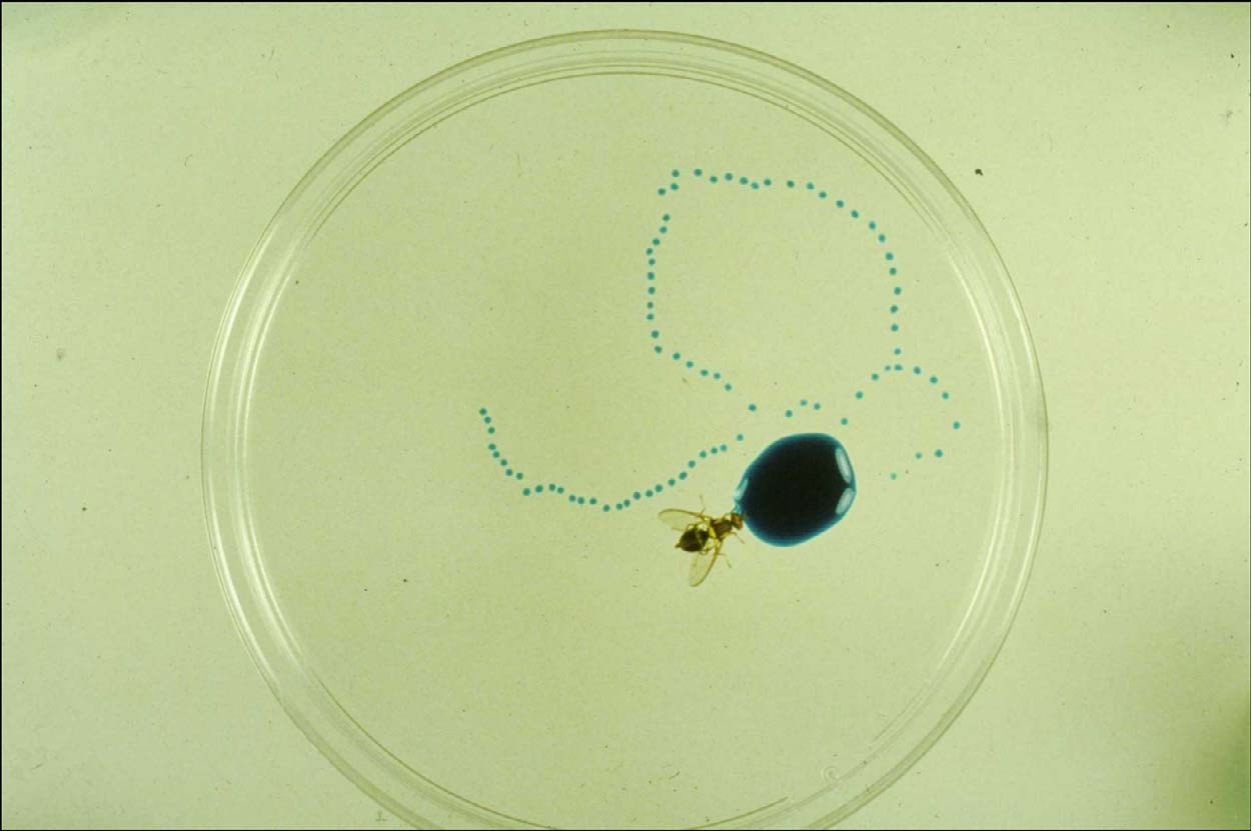
Regurgitation behaviour by *Bactrocera tyroni* - a long line of droplets has been regurgitated onto the resting surface following feeding on dilute sucrose solution coloured with non-toxic blue food dye.

**Figure 12.**
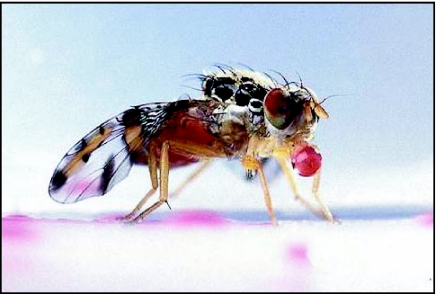
Post-feeding bubbling behaviour by *Ceratitis* *capitata* — a drop of liquid regurgitated from the crop is held between the labellar lobes. The fly had been fed dilute sucrose solution coloured with non-toxic red food dye.

Following feeding on a dilute solution of sucrose, hydrolysed yeast or natural fruit juices, adults of *Bactrocera tryoni* (Drew et al. 1983, Drew and Lloyd 1987) and *Anastrepha* species regurgitate a line of droplets on the feeding surface (Figure 11). The regurgitated droplets are reimbibed and the whole process repeated several times. In addition, adult flies also hold a large drop of regurgitated liquid between the everted lobes of the labellum, reingest it and repeat the process many times (Figure 12). Hendrichs et al. (1992) referred to this process as bubbling and demonstrated that adult *Rhagoletis* performed bubbling to concentrate crop contents by oral evaporation of excess water.

Secondly, the spaces between the pseudotracheal rings are about 0.5 µm in size, meaning that only fluids and particles less than 0.5 µm in size are ingested into the pseudotracheal lumen (Figure 8). The labellum is also flexed in a manner similar to *Bactrocera* species (see Figure 13 in Vijaysegaran et al. 1997) such that the oral opening is never exposed to the feeding substrate and the prestomal spines interlock and prevent the entry of large particles directly into the food canal (Figure 10). The diameter of the opening from the food canal into the pharynx is about 35–40 µm in size (Figure 2), making it physically impossible for a fly to ingest particles larger than this even if it were to fully evert the oral lobes (as do blow-flies) to feed.

In all of the feeding experiments where adults were fed with a range of particles ranging in size from less than 0.5 µm to about 40 µm in size, only particles < 0.5 µm in size were observed in the crops of flies. Thus, filtering of ingested fluids occurs in *C*. *capitata* as in the genus *Bactrocera*. Bacteria belonging to the family Enterobacteriaceae are less than 0.5 µm in size and thus are ingested along with fluids. Diazotrophic bacteria in the family Enterobacteriaceae, particularly the genera *Klebsiella*, *Citrobacter*, *Enterobacter* and *Pectobacterium*, have been shown to form a major fraction of the microbial community of the *C*. *capitata* gut and play a major role in nitrogen fixation in the gut (Behar et al. 2005). In the genus *Bactrocera*, Enterobacteriaceae have been also commonly isolated from the crop and gut, and these bacteria have been identified as *Klebsiella oxytoca*. *Erwinia herbicola* and *Enterobacter cloaceae* (Lloyd et al. 1986; Drew and Lloyd 1987) and less commonly *Serratia* spp., *Citrobacter fruendii*, *Proteus* spp., *Providencia rettgeri* and *Eschenchia coli* (Drew and Lloyd 1989). Drew et al. (1983) demonstrated that Enterobacteriaceae derived from plant surfaces provide some species of *Bactrocera*, including the Queensland fruit fly, *Bactrocera tryoni*, with a diet that enables sexually immature flies to reach sexual maturity and to reproduce. Subsequent work (Drew and Lloyd 1987) showed that adult *B*. *tryoni* and *B*. *neohumeralis* inoculate fruit surfaces with Enterobacteriaceae while foraging there. These bacteria then multiply (presumably on fruit surface leachates) and attracted immature flies, which in turn fed on these bacteria and attained sexual maturity. Thus, there is a growing body of evidence to suggest that the structural modifications on the labellum of fruit flies belonging to the genera *Ceratitis* and *Bactrocera* provide an elaborate labellar filtering mechanism that enables them to filter out yeasts, fungal spores and pollen that would be commonly found on the phylloplane, and feed selectively on Enterobacteriaceae in nature.

Such a labellar filtering mechanism in *Ceratitis* and *Bactrocera* species has important implications for field control of fruit flies using poisoned food baits and may help to explain why certain microbial pesticides such as *Bacillus thuringiensis* (Bt) have not been successfully used against tephritid pests. The micropores on the pseudotracheae of the adult fly only allow ingestion of fluids and particles less than 0.5 µm in size, whereas the average size of Bt spores is 2 µm in length and slightly less than 1 µm in width (Plomp et al. 2005), thus making the spores too large to be ingested through the micropores. The use of microbial pesticides in bait sprays may be successful for tephritids if the toxicant added to the bait dissolves completely into a liquid, or if the particle size of the toxicant in the formulation does not exceed 0.5 µm in size. This aspect of utilization of microbial and other biological agents as toxicants for pest tephritids has not been investigated before and could open up the use of newer and safer biological agents in bait sprays.

With regard to the structure of the labellum, *C*. *capitata*, however, differ from the *Bactrocera* in that the pseudotracheal rings have prominent blade-like projections that could be used to scrape or scarify the feeding substrate. Tephritids belonging to the genus *Blepharoneura* have pseudotracheal ring tips that have been modified into brushes and prominent blades. Adult *Blepharoneura* use these blades to cut and feed upon the tissues of their larval host plants (Driscoll and Condon 1994). In our feeding experiments in the laboratory, however, *C*. *capitata* were not observed scarifying or cutting into the feeding substrate. The blade-like projections in *C*. *capitata* are not as well developed as they are in *Blepharoneura*. Driscoll and Condon (1994) hypothesized that the blade-like projections in *Ceratitis* species are an ancestral condition and are not used to abrade the plant surface as do *Blepharoneura* species. The families Ulidiidae and Platystomatidae that also have blade-like ring tips on their pseudotracheae also do not use them to abrade the plant surface (Driscoll and Condon 1994). *C*. *capitata*, although possessing blade-like ring tips, was not observed to abrade the feeding surface, supporting the hypothesis of Driscoll and Condon (1994).
